# Prospective association between descriptive accelerometer-derived physical behaviour metrics and cardiometabolic risk indicators in Dutch children: The ABCD study

**DOI:** 10.1016/j.jesf.2025.200431

**Published:** 2025-11-26

**Authors:** Fawad Taj, J.M.M. Chinapaw, Teatske Altenburg, Tanja Vrijkotte

**Affiliations:** a Amsterdam UMC location Vrije Universiteit Amsterdam, Public and Occupational Health, De Boelelaan 1117, Amsterdam, the Netherlands; b Amsterdam Public Health, Health Behaviors and Chronic Diseases, Amsterdam, the Netherlands; c Amsterdam Public Health, Methodology, Amsterdam, the Netherlands; d Amsterdam Public Health, Digital Health, Amsterdam, the Netherlands; e Department of Public and Occupational Health, Amsterdam UMC, Location University of Amsterdam, Amsterdam, the Netherlands; f Amsterdam Reproduction and Development Research Institute, Amsterdam, the Netherlands

**Keywords:** Accelerometer, Count-based metrics, Physical activity, BMI-SDS, MetScore, Sedentary behaviour, MVPA, Cardiometabolic risk

## Abstract

To better understand which aspects of physical behavior are most critical for health, this study investigated prospective associations between various accelerometer-derived physical activity (PA) and sedentary behaviour (SB) metrics with cardiometabolic risk indicators. We analyzed waist-worn accelerometer data from 114 Dutch children (50 girls), age 8.6 ± 0.4 years, in the Amsterdam Born Children and Development (ABCD) cohort. Physical behavior metrics included volume-based metric (counts per minute [CPM]), time-use metrics (e.g., time spent in different physical behaviour intensities), pattern-based metrics (e.g., fragmentation indices), and intensity gradient. Health indicators, assessed at age 11–12 years, included BMI-standard deviation score (SDS) and a cardiometabolic risk score (MetScore) calculated from BMI, systolic and diastolic blood pressure, high-density lipoprotein, total cholesterol, and triglycerides. Regression analyses were conducted to examine associations between physical behaviour metrics and both BMI-SDS and MetScore.

Linear regression analyses, adjusted for gender and wear time, revealed that more time spent in moderate-to-vigorous physical activity (MVPA), a conventional time-use metric, was significantly associated with lower BMI-SDS (β = −0.02; 95 % CI: 0.03, 0), but not with MetScore. Other physical behavior metrics, including volume-based, pattern-based, and intensity gradient measures, did not show significant associations with either BMI-SDS or MetScore in this cohort. These findings indicate that conventional time-use metrics, particularly MVPA, may be more effective in detecting health associations in small homogenous populations. Future research with larger populations is necessary to determine if alternative metrics provide additional insight into the association between physical behavior and cardiometabolic health.

## 1 Introduction

Cardiometabolic risk factors, including adiposity, elevated cholesterol levels, hypertension, and impaired glucose metabolism, are well-established precursors to cardiovascular diseases (CVD) and premature mortality.^1^ These risk factors often emerge during childhood and adolescence, with a tendency to persist into adulthood, thereby contributing to the development of CVD later in life.^2^ Physical behaviours, such as physical activity (PA) and sedentary behaviour (SB), play a crucial role in shaping cardiometabolic health during these formative years.^2^, ^3^ Understanding how these physical behaviours influence cardiometabolic risk profiles early in life is essential for developing effective strategies to prevent CVD and improve long-term population health outcomes.

Accelerometers are increasingly used to estimate different types and aspects of physical behaviours, e.g. types (walking, running, cycling etc.) and intensities (sedentary, light, moderate and vigorous PA).^4^ In epidemiology and public health research, the focus is often on time-use in certain behaviour, which rely on cut-points to classify physical activity into categories such as sedentary, light, moderate, and vigorous activity.^5^ However, cut-point-based approaches have limitations, as they may not fully capture the complexity of movement patterns, because inherently it can underestimate overall activity exposure because period not meeting threshold are excluded, meaning that much of the data is discarded and the full distribution of movement intensities is not represented. To address this, cut-point-free metrics have been introduced, offering a more comprehensive representation of physical behavior.^6^, ^7^, ^8^, ^9^, ^10^

To establish a standardized framework for defining and analyzing physical behaviour metrics, the GRANADA consensus.^11^ categorized a range of descriptive metrics and analytical approaches for assessing associations with accelerometer-derived physical behaviours in epidemiological research. Among these metrics, we selected four classes of metrics because the represent the main domain of accelerometer-deriver physical behaviours, together, they capture overall movement, time distribution, behavior patterns and the intensity spectrum, which align with our objective of comprehensively examining prospective association with children's cardiometabolic health.

First, the volume-based metrics, such as average acceleration/counts or steps per minute, estimate total PA-related energy expenditure and serve as straightforward proxies for overall activity levels and energy expenditure.^7^^,^^11^ Second, the most common and mostly used time-use metrics quantify the duration of specific activities, such as time spent in SB or moderate-to-vigorous physical activity (MVPA). Third, pattern-based metrics, such as the fragmentation index, quantify interruptions or transitions across various intensities, thereby offering insights into the rhythmic patterns of physical behaviours.^12^ Higher fragmentation indices indicate more frequently interruption in sedentary or MVPA bouts, which is considered beneficial for SB (breaking up prolonged sitting) but less favorable for MVPA (failure to sustain activity). Lastly, the Intensity Gradient (IG) reflects the distribution of activity across the intensity spectrum; a shallower (less negative slope indicates greater accumulation of mid-to-high intensity activity. IG is derived by sorting the full intensity spectrum of accelerometer/counts data into bins based on a specific intensity and the time accumulated at each intensity and thus reflecting both volume and intensity.^13^ The intensity gradient is the curvilinear relationship between intensity and time spent in each successive time bin between 0 and 4000 mg, transformed to a linear relationship using the natural log of each variable.^13^

Previous studies investigating cut point-free metrics in children have reported that higher physical activity intensity, as represented by IG, was negatively associated with BMI z-score, independent of total activity volume.^7^^,^^14^ Evidence regarding time-use and pattern-based metrics is more limited, but emerging studies suggest that prolonged sedentary bouts and lower fragmentation of activity are linked with less favorable cardiometabolic profiles.^15^^,^^16^ Notably, most studies on physical activity behavior and cardiovascular health focuses primarily on adiposity outcomes such as BMI, while relatively few have examined broader cardiometabolic profiles encompassing multiple physiological systems.

To address this gap, the present study focuses on two complementary cardiometabolic risk indicators: BMI-standard deviation score (BMI-SDS) and a composite cardiometabolic summary score (MetScore). The BMI-SDS, equivalent to the BMI z-score in this context, represents age- and sex-adjusted adiposity based on Dutch growth references and is widely used and validated in pediatric research. In contrast, cardiometabolic summary score (MetScore) was included to capture a broader constellation of cardiometabolic risk factors including blood pressure, lipid profiles and triglycerides, derived through confirmatory factor analysis (CFA) to represent an integrated latent construct of risk.^17^ MetScore via CFA offers two key advantages: it integrates multiple cardiometabolic components into a single latent construct while weighting them according to their shared variance, thereby reducing measurement error and avoiding equal weighting of heterogeneous factors as in z-score sums.

Traditional cardiometabolic risk scores commonly estimate overall health risk by aggregating multiple physiological and clinical risk factors into a single measure. However, recent literature highlights the limitations of using such aggregated measures, such as the sum or standardized z-scores of cardiometabolic risk factors as indicators of overall risk.^18^ For example, research has shown that specific lipid subfractions, such as small LDL and HDL particles, can have different impacts on cardiovascular risk.^19^ Despite identical aggregated z-scores, their actual cardiovascular risks differ, underscoring how this approach oversimplifies the distinct contributions of individual risk factors.^18^ By deriving the MetScore through CFA, we address these limitations and obtain a statistically robust latent construct representing overall cardiometabolic risk. By considering both these risk indicators, we aimed to examine whether associations with physical behaviour metrics differ depending on whether risk is operationalized through a single anthropometric measure or through a multidimensional composite index.

Therefore, the present study aimed to examine the prospective associations between accelerometer-derived physical behaviour metrics, including volume-based, time-used, pattern-based, and intensity-based and two complementary cardiometabolic risk indicators (BMI-SDS and MetScore) in Dutch children from the Amsterdam Born Children and their Development (ABCD) cohort.

## 2 Methods

### 2.1 Study sample

**Fig. 1:**
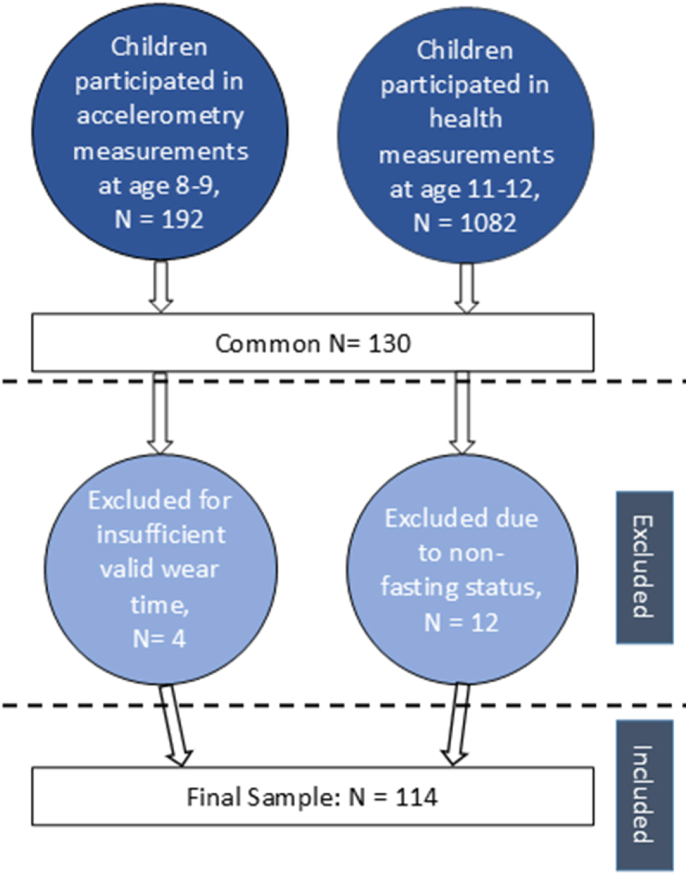
Overview of study sample. Insufficient wear time refers to participants who did not meet the minimum valid wear-time criteria (≥8 h/day on at least 3 weekdays and 1 weekend day).

This study is based on data from the Amsterdam Born Children and their Development (ABCD) birth cohort,^20^ which was established to assess the long-term impact of prenatal and early-life influences on later health.^20^, ^21^ The current analysis utilized accelerometry data, collected when children were age 8–9 years (September/October 2012), and cardiometabolic health data collected at age 11–12 years (2015–2016).^21^, ^22^ Fig. 1 illustrates the derivation of the analytical sample. Among the 192 children who provided valid accelerometer data at age 8–9 years and the 1082 who participated in cardiometabolic health assessments at age 11–12 years, 130 had data available from both time points. Of these, four were excluded for not meeting the minimum valid wear-time criteria, and twelve were excluded due to non-fasting status during blood collection, which could affect the accuracy of biochemical measures used to compute the MetScore. The final analytical sample therefore included 114 children with complete accelerometer and fasting cardiometabolic data.

### 2.2 Physical behaviour (age 8–9 years)

Physical behaviours were assessed using triaxial ActiTrainers (dimensions: 8.6 cm × 3.3 cm × 1.5 cm, weight: 51 g) or GT3Xs (dimensions: 3.8 cm × 3.7 cm × 1.8 cm, weight: 27 g), both produced by ActiGraph LLC and widely validated in pediatric studies. Previous research indicates that while ActiTrainer and Gt3X accelerometers can yield broadly comparable estimates of MVPA,^23^ although small systematic difference in raw activity counts across models have also been reported.^24^ In this study, data from both devices were initiated in ActiLife software (version 6.13.3) at a sampling frequency of 30Hz and harmonized within ActiLife prior to further processing to reduce inter-device variability. Nonetheless, the use of two devices model represents a limitation, as some residual differences in output may persist.

Researchers distributed the accelerometers face-to-face, providing verbal and written information about accelerometer use to both children and one of their parents. As part of the accelerometer wear protocol, researchers placed the devices on the children's waist using an elastic waistband and instructed them to wear the device for at least seven consecutive days during waking hours, removing it only for sleeping, swimming, and bathing.^21^

Additional data processing criteria is applied using Actilife software, periods of more than 60 min of consecutive zero counts were defined as non-wear time and excluded from the analysis.^25^ An epoch length of 15 s was used, consistent with recommendations for pediatric populations as it balances between sensitivity to intermittent nature of children's physical activity patterns and minimizing data burden.^26^^,^^27^ In addition, the ABCD cohort accelerometer data had previously been processed using a 15-s epoch, allowing us to ensure methodological consistency and directly compare our findings with earlier studies based on the same dataset. Days with at least 8 h of valid wear time were considered and children with at least three valid weekdays and one valid weekend day were included in further data analysis.

All metrics were derived from data recorded by the vertical axis. Because accelerometers do not directly capture posture, sedentary behaviour represents inactivity used as a proxy for sitting or lying time. A count <25 per 15 s was used to define sedentary behaviour time and count ≥574 per seconds was used for MVPA time. These are the among the most validated and recommended cut-points for youth physical activity assessment using accelerometry,^28^, ^29^^,^^30^ and also, the ABCD cohort accelerometer data had previously been processed using a 15-s epoch, allowing us to ensure methodological consistency and directly compare our findings with earlier studies based on the same dataset.^21^ To derive fragmentation index metrics, a sedentary bout was identified as any period of at least 10 consecutive minutes with counts <25 per epoch with zero tolerance, and an MVPA bout was defined as a period of at least five consecutive minutes with counts ≥574 per epoch and an absolute tolerance of 30 s.^31^

Using the above defined thresholds, metrics describing volume, time-use, patterns, and intensity gradients of physical behaviours were calculated using a customized R script. Table 1 provides the categorization and detailed descriptions of each metric.

**Table 1:** Descriptions/definition of physical behaviour metrics.

Category	Metrics	Details
Time-use metrics	Time spent (min/day) in SB and MVPA	SB < 25 counts/15 s
		MVPA ≥574 counts/15 s
Volume-based metric	Counts per minute (CPM)	Arithmetic average of the processed acceleration (counts) throughout the measurement period
Pattern-based metrics	SB and MVPA Fragmentation Indexes	Ratio between the number of bouts of a specific intensity to the total time spent at that intensity.^12^, ^32^
Intensity-gradient	Intensity Gradient (Count-Based)	Count-based IG was calculated using a resolution of 25 counts per bin, with the final output encompassing a total of 361 bins.

### 2.3 Cardiometabolic risk indicators

Height and weight were measured using a portable Leicester stadiometer (Seca, Hamburg, Germany) and a Marsden weighing scale (Model MS-4102, Rotherham, United Kingdom), respectively.^33^ Body Mass Index (BMI, kg/m^2^) was calculated as an individual's weight (in kilograms) divided by the square of their height (in meters). To account for age- and sex-specific variability, BMI was standardized using LMS tables, which incorporate three parameters: Lambda (L) for skewness, Mu (M) for the median, and Sigma (S) for the generalized coefficient of variation.^34^ The reference values for standardization were derived from a comprehensive Dutch nationwide growth study^17^^,^^35^

Blood pressure (BP) was measured in a lying position using the Omron 705 IT device (Omron Healthcare Inc., Bannockburn, IL, USA) with a small cuff. Two BP measurements were taken after 5 min of rest, and if the difference between measurements exceeded 10 mm Hg, a third measurement was taken. The average of the two closest systolic and diastolic BP readings was used for analysis Krijger et al., 2021.

Capillary blood samples were collected by finger-prick after 3 h of fasting. Samples were analyzed using an Alere Cholestech LDX Analyzer (Alere Inc., Abbott, Chicago, IL, USA). Blood analysis included High-Density Lipoprotein (HDL), total cholesterol, triglycerides, and fasting glucose.^33^

**Fig. 2:**
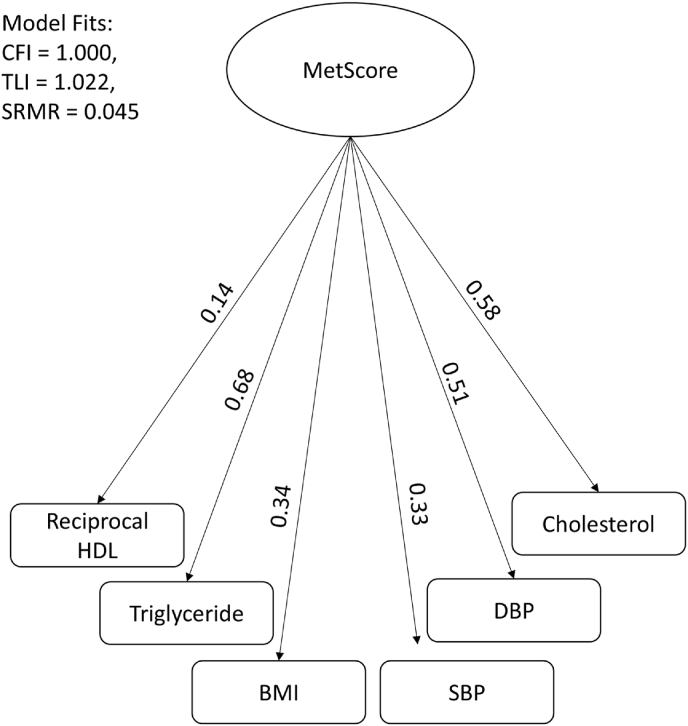
Factor loadings for cardiometabolic risk factor clustering (MetScore). All components are standardized into z-scores. Abbreviations: CFI = Comparative Fit Index, TLI = Tucker-Lewis Index, SRMR = Standardized Root Mean Square Residual. BMI= Body Mass Index, SBP= Systolic Blood Pressure, DBP = Diastolic Blood Pressure, HDL= High-Density Lipoproteins.

We calculated the MetScore using confirmatory factor analysis (CFA) representing six cardiometabolic risk factors: total cholesterol, HDL, triglycerides (TG), BMI, SBP, and SBP.^17^, ^34^, ^35^ TG and total cholesterol were standardized using a log transformation, followed by z-score standardization to achieve a mean of 0 and a standard deviation of 1. After standardization of HDL, inverse transformation was performed (1/HDL), because HDL is a good cholesterol. The model achieved an excellent fit, as evidenced by the Comparative Fit Index (CFI = 1.000) and Tucker-Lewis Index (TLI = 1.022), both exceeding the standard threshold of 0.95 (see Fig. 2). The Standardized Root Mean Square Residual (SRMR = 0.045) indicated minimal error in model fit, further supported by a non-significant chi-square test (χ^2^(5) = 4.267, p = 0.512).

### 2.4 Statistical analyses

All statistical analyses were performed using R (version 4.3.2) language in RStudio 2023.12.1 + 402 for windows. We calculated the descriptive statistics (mean ± SD) for physical behaviour metrics and cardiometabolic indicators, stratified by gender to characterize the study population. These values are descriptive only and were not formally tested for statistical differences, as gender was included as a covariate in all regression models. Separate linear regression models were developed to investigate the associations between CPM, time spent in SB and MVPA (min/day), SB and MVPA fragmentation index, and intensity gradient at age 8–9 years, with primary outcome variables BMI-SDS and cardiometabolic risk scores (MetScore) at age 11–12 years. Standardized regression coefficients (β) and 95 % confidence intervals (CI) were reported for all models, with p-values <0.05 considered statistically significant.

All analyses were adjusted for wear time and gender, as these were the most influential factors in ABCD cohort. Other potential covariates, such a socioeconomic status and ethnicity showed little to no variability because the ABCD sample consisted almost entirely of Amsterdam-born DUTCH children from similar sociodemographic backgrounds. Including these would therefore not have added explanatory power and could have increased the risk of model overfitting given the modest sample size. To address the interdependency between SB and MVPA time, the analysis model for SB and MVPA time were adjusted for MVPA time and vice versa. We assessed multicollinearity among independent variables using variance inflation factors (VIF). All VIF values were below 5, indicating no significant multicollinearity.^36^ Model fit was evaluated using adjusted R^2^ values, with negative values indicating poor fit.

No a priori power analysis was conducted. Post-hoc power calculations for linear regression indicated that with N = 114, the study had limited power (<10 %) to detect small-to-moderate effect sizes (Cohen's f^2^ = 0.02–0.15). This suggests that the study was adequately positioned to detect only strong associations, while weaker associations, particularly those involving composite outcomes such as MetScore, may not have been detectable.

Furthermore, Sensitivity analyses were conducted to assess the robustness of the results. Residual diagnostics evaluated the model assumptions and identified potential outliers and influential observations. Observations with Cook's distance exceeding 0.5 or showing unusual residual patterns were flagged for further investigation. Six Instances were identified as extreme values based on these diagnostics.

## 3 Results

Table 2 shows the descriptive statistics for physical behaviour metrics and cardiometabolic indicators stratified by gender (boys (N = 64) and girls (N = 50). These values are intended to characterize the sample distribution, while gender differences were formally accounted for as a covariate in regression models. Overall, boys spent more time in MVPA (43.40 ± 17.20 min/day), less time sedentary (422.00 ± 48.40 min/day) and had lower counts per minute (624.00 ± 137.00 counts/min) than girls (29.00 ± 10.40 min/day MVPA, 432.00 ± 55.60 min/day SB and 525.00 ± 128.00 counts/min, respectively).

**Table 2:** Characteristics (mean (SD)) of participants: physical behaviour metrics, health measurements and cardiometabolic risk indicators.

	Boys (N = 64)	Girls (N = 50)	Overall (N = 114)
**Physical behaviours at age 8**–**9 years**			
Wear Time (min/day)	751.00 (30.80)	742.00 (48.60)	747.00 (39.70)
**Volume-based metrics**			
Average CPM (counts/min)	624.00 (137.00)	525.00 (128.00)	580.00 (142.00)
**Time-used metrics**			
Sedentary Time (min/day)	422.00 (48.40)	432.00 (55.60)	426.00 (51.70)
MVPA Time (min/day)	43.40 (17.20)	29.00 (10.40)	37.00 (16.20)
**Pattern-based metrics**			
SB Fragmentation Index	0.07 (0.01)	0.06 (0.01)	0.07 (0.01)
MVPA Fragmentation Index	1.44 (1.12)	0.78 (0.62)	1.22 (1.02)
**Intensity Gradient (slope)**	−1.95 (0.13)	−1.99 (0.15)	−1.97 (0.14)

**Health measurements at age 11–12 years**			
Weight (kg)	39.80 (6.80)	41.90 (9.20)	40.70 (7.98)
Height (cm)	150.00 (6.22)	152.00 (6.69)	151.00 (6.25)
Total Cholesterol (mmol/L)	4.04 (0.65)	4.11 (0.60)	4.07 (0.63)
HDL (mmol/L)	1.49 (0.32)	1.46 (0.28)	1.47 (0.30)
Triglycerides (mmol/L)	1.04 (0.67)	0.98 (0.44)	1.01 (0.57)
Body Mass Index (kg/m^2^)	16.90 (1.96)	17.50 (2.51)	17.20 (2.23)
SBP (mmHg)	106.00 (7.38)	106.00 (8.64)	106.00 (7.92)
DBP (mmHg)	60.30 (6.40)	59.30 (5.91)	59.80 (6.18)

Cardiometabolic risk **indicators at age 11–12 years**			
BMI-SDS	−0.46 (0.89)	−0.42 (1.07)	−0.44 (0.97)
MetScore	−0.02 (0.52)	0.03 (0.41)	0.00 (0.47)

Fragmentation indices and intensity gradients were generally similar between boys and girls. However, boys had a higher MVPA fragmentation index (1.44 ± 1.12) than girls (0.78 ± 0.62), indicating that girls’ MVPA was accumulated in shorter bouts than boys.

Table 3 shows the results of the associations between physical behaviour metrics and both BMI-SDS and MetScore. MVPA time had a small but significant association with BMI-SDS (β = −0.02; 95 % CI: 0.03, 0, p < 0.05), whereas the other metrics did not exhibit significant association with either BMI-SDS or MetScore.

**Table 3:** Association between physical behaviour metrics with BMI-SDS and MetScore. Abbreviations: SDS: standardized Deviation score, SB: Sedentary, MVPA: Moderate-to-vigorous physical activity.

	Category	Model	BMI-SDS β (95 % CI)	MetScore β (95 % CI)
1	Time-used metrics	Sedentary Time	0.00 (−0.01, 0.00)	0.00 (−0.01, 0.00)
2		MVPA Time	−0.02 (−0.03, 0.00)^a^	0.00 (−0.01, 0.00)
3	Volume-based metrics	Counts Per Minute (CPM)	0.00 (0.00, 0.00)	0.00 (0.00, 0.00)
4	Pattern-based metrics	SB Fragmentation Index	−11.01 (−28.03, 6.01)	0.85 (−6.23, 7.93)
5		MVPA Fragmentation Index	−0.03 (−0.31, 0.24)	−0.10 (−0.26, 0.06)
6	Intensity Gradient (slope)	Intensity Gradient	−0.39 (−1.71, 0.93)	−0.22 (−0.91, 0.48)

a Show the statistically significant results (p > 0.05).

The sensitivity analyses, after excluding around 11% of the extreme observation from the total observations, revealed major adjustment in SB fragmentation index for both BMI-SDS (β = −9.69; 95 % CI: 23.93, 4.55) and MetScore (β = 1.16; 95 % CI: 5.09, 7.41), but still not reached the statistically significant level. Moreover, a minor adjustment in estimates and significance levels for MVPA time-use in both BMI-SDS (β = −0.02; 95 % CI: 0.04, −0.01) and MetScore (β = −0.01; 95 % CI: 0.02, 0.00) were observed. Metrics that capture both pattern and volume dimensions, such as the fragmentation indices and intensity gradient, did not demonstrate stronger associations with BMI-SDS or MetScore compared to traditional volume-based metrics.

## 4 Discussion

This study investigated the prospective associations between accelerometer-derived physical behaviour metrics and cardiometabolic risk indicators - BMI-SDS and MetScore in children from the ABCD cohort. Among the physical behavior metrics examined, only MVPA at age 8–9 years was prospectively associated with lower BMI-SDS at age 11–12 years and no association was observed between MVPA and MetScore.

Our findings align with prior studies reporting weak or inconsistent associations between physical behaviour metrics and BMI in children, particularly in highly active and normal weight samples^37^, ^38^ Whereas, the studies using cut-point-free metrics, such as average acceleration and intensity gradient, have reported links with BMI in some cohorts.^14^ Our cohort consisted of predominantly healthy and active Dutch children, which likely reduced variability in both physical behaviours and cardiometabolic outcomes.^39^, ^40^, ^41^ This homogeneity may explain why only MVPA was associated with BMI-SDS, consistent with the law of diminishing returns, where health benefits are less pronounced once baseline activity levels are already high. Future studies should investigate fewer active populations and apply more specific high-intensity metrics, such as vigorous activity, or identify peak activity periods (e.g., most active 5 or 10 min), to better understand health enhancements in already active groups.

A key strength of this study lies in its prospective design, which utilized advanced statistical methods such as factor analysis to derive a latent cardiometabolic risk score (MetScore) and BMI-SDS. These scores enabled a nuanced exploration of the relationships between physical behaviors and cardiometabolic health. Furthermore, cut-point-free metrics, such as the intensity gradient, have been promoted as reflecting the principle that ‘every movement counts,^42^ because they capture the full spectrum of movement intensities rather than discarding activity below a threshold. In contrast, cut-point-based metrics like MVPA ignore lower-intensity activity. However, in our relatively healthy and active cohort, only MVPA showed significant associations with BMI-SDS, while cut-point-free metrics did not. This suggests that although cut-point-free metrics conceptually align with the idea that all movement contributes to health, their added value may become more apparent in less active or more heterogeneous populations. The lack of universal cut-off values for defining movement interruptions and inactivity periods may explain why fragmentation indices were not directly related to BMI-SDS as a measure of adiposity.^43^

Importantly, MVPA was not associated with MetScore, reinforcing that body composition (BMI-SDS) and aggregated cardiometabolic risk capture distinct health dimensions. The MetScore, while advantageous in integrating multiple biomarkers through confirmatory factor analysis, may require larger and more heterogeneous samples to reveal meaningful associations. This analytical distinction supports our decision to examine BMI-SDS and MetScore separately. Furthermore, the findings suggest that in relatively healthy, active child populations, interventions may need to emphasize maintenance or enhancement of MVPA to support healthy weight trajectories. At the public health level, this highlights the importance of ensuring children meet daily MVPA recommendations. Clinically, accelerometer-derived metrics such as time-spent in MVPA may be the most practical for monitoring and feedback, given their demonstrated sensitivity to health outcomes in this context.

Despite its strengths, this study has limitations that warrant consideration. The relatively small and homogeneous sample of primarily healthy-weight, ethnically Dutch children may limit the generalizability of findings to more diverse populations. We were unable to report the total number of children invited or consented for accelerometer participation, as these data were collected in 2012 and were not available in detail. Our analyses therefore reflect a subset of the ABCD cohort with complete exposure and outcome data. Our modest sample size limited statistical power, particularly for regression analyses with multiple predictors. As a result, weaker associations may have gone undetected, and findings regarding null results (especially for MetScore) should be interpreted cautiously. Future research should aim to replicate these analyses in larger, more heterogeneous cohorts to better understand the role of physical behavior metrics across different demographic groups.

## 5 Conclusion

In this study, we examined the prospective associations between various aspects of physical behaviour in Dutch children aged 8–9 years, measured using both cut-point-based and cut-point-free accelerometer metrics and cardiometabolic health outcomes at ages 11–12 years, represented by BMI-SDS and MetScore. Among the different metrics investigated, only the conventional time-use measure of moderate-to-vigorous physical activity (MVPA) showed a significant inverse association with BMI-SDS, whereas no associations were observed with MetScore. Other metrics, including volume-based (e.g., counts per minute) and pattern-based indicators (e.g., fragmentation indices) and intensity gradients, were not significantly related to either outcome.

These findings suggest that traditional time-use metrics remain the most sensitive indicators of adiposity in relatively healthy and homogeneous child populations. The absence of associations with MetScore indicates that anthropometric and biochemical markers may capture distinct dimensions of cardiometabolic health. Methodologically, the study demonstrates the feasibility of incorporating cut-point-free accelerometer metrics into prospective cohort analyses, although larger and more diverse samples are required to fully elucidate their potential utility.

From a public-health perspective, strategies should continue to promote daily MVPA in children, while future research should investigate whether higher-intensity or more sustained activity patterns provide additional benefits in less active populations. Given the complexity of physical activity's relationship with cardiometabolic health, future studies should employ larger and more heterogeneous cohorts and consider deriving metrics from raw g-based accelerometer data rather than traditional count-based outputs to enhance precision and comparability across studies.

## Author contributions

Dr. Fawad Taj conceived the study, conducted the data analysis, and drafted the initial manuscript. Prof. J.M.M. Chinapaw, Dr. Teatske Altenburg, and Dr. Tanja Vrijkotte contributed to the study design, data interpretation, and critical revision of the manuscript. All authors reviewed and approved the final version of the manuscript.

## Ethical approval and consent to participate

The Amsterdam Born Children and their Development (ABCD) study was approved by the Medical Ethics Committee of the Amsterdam UMC. Written informed consent was obtained from the parents or legal guardians of all participating children.

## Funding

This research did not receive any specific grant from funding agencies in the public, commercial, or not-for-profit sectors.

## Declaration of competing interest

The authors declare that they have no known competing financial interests or personal relationships that could have influenced the work reported in this paper.
