# Corrigendum to “Prospective association between descriptive accelerometer-derived physical behaviour metrics and cardiometabolic risk indicators in Dutch children: The ABCD study” [J Exerc Sci Fitness 24 (2026) 200431]

**DOI:** 10.1016/j.jesf.2026.200455

**Published:** 2026-02-13

**Authors:** Fawad Taj, Teatske M. Altenburg, Tanja G.M. Vrijkotte, Mai J.M. Chinapaw

**Affiliations:** a Amsterdam UMC Location Vrije Universiteit Amsterdam, Public and Occupational Health, De Boelelaan 1117, Amsterdam, the Netherlands; b Amsterdam Public Health, Health Behaviors and Chronic Diseases, Amsterdam, the Netherlands; c Amsterdam Public Health, Methodology, Amsterdam, the Netherlands; d Amsterdam Public Health, Digital Health, Amsterdam, the Netherlands; e Department of Public and Occupational Health, Amsterdam UMC, Location University of Amsterdam, Amsterdam, the Netherlands; f Amsterdam Reproduction and Development Research Institute, Amsterdam, the Netherlands

**The authors regret:**

In the originally published version of this article, the order of authors was incorrect. The correct author order is shown above.

Additionally, the funding information was inadvertently omitted. **The correct** funding **statement is:**

“This research is part of the SmartCHANGE project (GA No.101080965), ‘AI-based long-term health risk evaluation for driving behaviour change strategies in children and youth,’ funded by a grant from Horizon Europe – the Framework Programme for Research and Innovation (HORIZON-HLTH-2022-STAYHLTH-01-04). The funders had no role in study design, data collection and analysis, decision to publish, or preparation of the manuscript.”

**The authors would like to apologise for any inconvenience caused.**

